# A Multi-Disulfide Receptor-Binding Domain (RBD) of the SARS-CoV-2 Spike Protein Expressed in *E. coli* Using a SEP-Tag Produces Antisera Interacting with the Mammalian Cell Expressed Spike (S1) Protein

**DOI:** 10.3390/ijms23031703

**Published:** 2022-02-01

**Authors:** Subbaian Brindha, Yutaka Kuroda

**Affiliations:** Department of Biotechnology and Life Science, Faculty of Engineering, Tokyo University of Agriculture and Technology, 2-24-16 Nakamachi, Koganei-shi, Tokyo 184-8588, Japan; s197246x@st.go.tuat.ac.jp

**Keywords:** *Escherichia coli* expression, disulfide bond, solubility, fusion tag, immunogenicity

## Abstract

An *Escherichia coli* (*E. coli*) production of the receptor-binding domain (RBD) of the SARS-CoV-2 (isolate Wuhan-Hu-1) spike protein would significantly accelerate the search for anti-COVID-19 therapeutics because of its versatility and low cost. However, RBD contains four disulfide bonds and its expression in *E. coli* is limited by the formation of aberrant disulfide bonds resulting in inclusion bodies. Here, we show that a solubility-enhancing peptide (SEP) tag containing nine arginine residues (RBD-C9R) attached at the C-terminus can overcome this problem. The SEP-tag increased the expression in the soluble fraction and the final yield by five times (2 mg/L). The folding properties of the *E. coli* expressed RBD-C9R were demonstrated with biophysical characterization using RP-HPLC, circular dichroism, thermal denaturation, fluorescence, and light scattering. A quartz crystal microbalance (QCM) analysis confirmed the binding activity of RBD-C9R with ACE2, the host cell’s receptor. In addition, RBD-C9R elicited a Th-2 immune response with a high IgG titer in Jcl: ICR mice. The RBD-C9R antisera interacted with both itself and the mammalian-cell expressed spike protein (S1), as demonstrated by ELISA, indicating that the *E. coli* expressed RBD-C9R harbors native-like epitopes. Overall, these results emphasize the potential of our SEP-tag for the *E. coli* production of active multi-disulfide-bonded RBD.

## 1. Introduction

Severe acute respiratory syndrome Coronavirus 2 (SARS-CoV-2) [1] is responsible for the COVID-19 pandemic and continues to pose a global health threat, despite the availability of vaccines that are mRNA-based, vector-based, inactivated viruses, or DNA vaccines [2]. On the other hand, subunit protein vaccines also offer advantages in terms of cost, production capacity, transport, and administration [3].

SARS-CoV-2 is a single-stranded, positive-sense RNA virus belonging to the coronaviridae family [4]. SARS-CoV-2 is made of four major structural proteins [4], where the homotrimeric spike protein (S protein) mediates the viral entry into the host cells through the binding of the receptor-binding domain (RBD) with ACE2 (angiotensin-converting enzyme-2), the host receptor [5,6]. Specific binding of the RBD to ACE2 is essential for infection [7] and the RBD is thus a promising target for the design of therapeutics and the production of neutralizing antibodies [8].

The RBD of SARS-CoV-2 (isolate Wuhan-Hu-1) spans residues 319–541 of the spike protein. It is a β-sheet protein and contains four disulfide bonds and one free cysteine. Three disulfide bonds (Cys336/Cys361, Cys379/Cys432, Cys391/Cys525) help stabilize the β sheet structure and the fourth one (Cys480/Cys488) connects the loops in the distal end of the RBM (receptor-binding motif) [5]. RBD is currently expressed in eukaryotic expression systems, limiting its potential advantage as a vaccine candidate or target for drug development.

RBD expressed in *E. coli* could provide a fast and cost-effective production system. However, the expression of a multi disulfide protein in *E. coli* often results in non-native S-S bonds producing misfolded protein expressed in the inclusion body [9]. Fusion proteins such as thioredoxin (TRX), small ubiquitin-like modifiers (SUMO), maltose-binding proteins (MBP), and glutathione-S-transferase (GST) were used for expressing multi disulfide-bonded proteins [10] in a soluble form. However, their solubilizing effects are poorly predictable and often necessitate much trial and error. Furthermore, because of their large sizes, fusion proteins need to be removed from the target protein as they may interfere with its structure and activity [10].

Solubility enhancing peptide (SEP) tags are artificially designed 5 to 10 residue peptides made of a single or few types of amino acids attached to the protein termini [11,12,13]. They significantly increase the protein’s solubility without much affecting its structure or activity [14,15]. We previously used SEP-tags for improving the solubility and yield of recombinant proteins: EGFR-ECD-III [16], anti-EGFR-ScFv [17], TEV protease [18], and Gaussia luciferase [12].

Here, we demonstrate the ability of a SEP-tag, containing nine arginines (C9R) attached to the C-terminus of RBD (RBD-C9R; Molecular weight, 29.60 kDa), to improve the *E. coli* expression of RBD in a soluble form. The *E. coli* expressed RBD-C9R displayed interaction to human ACE2 and RBD-C9R antisera produced in mice recognized the commercial mammalian-cell expressed spike protein (S1).

## 2. Results

### 2.1. Plasmid Construction

A synthetic gene encoding SARS-CoV2 (isolate Wuhan-Hu-1) RBD (UniProt ID P0DTC2) was designed with codon optimization for *E. coli*. A SEP-tag, C9R, consisting of three repeated blocks of three arginine residues preceded by a glycine (GR_3_)_3_ was introduced at the C-terminus of the SARS-CoV2 RBD using a 2-glycine linker between SARS-CoV2 RBD and the C9R tag. The gene fragment was subcloned in the pET15b vector with a 6X histidine tag at the N-terminus. The untagged variant was constructed by introducing the stop codon just after the RBD sequence but before the C9R tag through site-directed mutagenesis yielding the untagged expression vector pET15b-poly (His)6-RBD. DNA sequencing results indicated that both tagged and untagged variants were successfully constructed (data not shown).

### 2.2. RBD Expression and Purification

**Figure 1:**
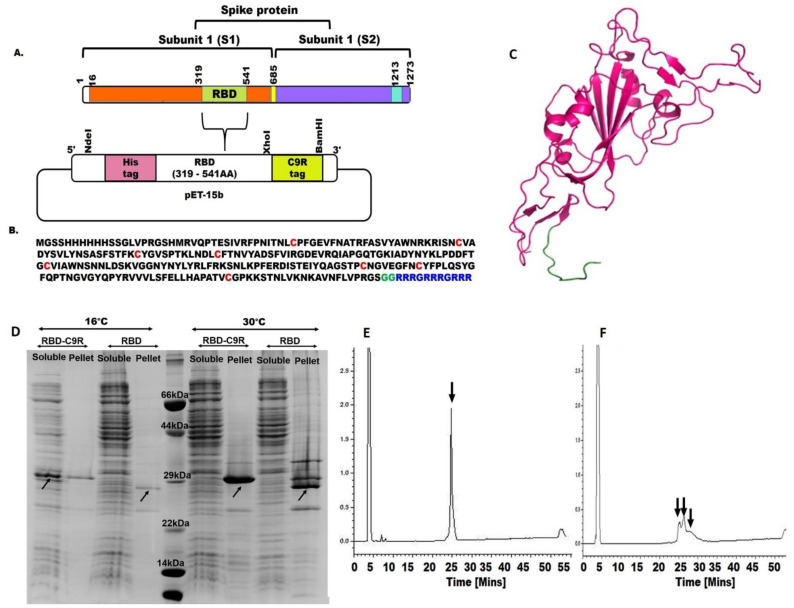
Strategy for cloning of SARS-CoV-2-C9R into pET15b vector. (**A**) Schematics of the sequence location of RBD in the SARS-CoV-2 spike protein and pET15b expression vector of RBD-C9R. (**B**) Amino acid sequence of the target gene cassette. (**C**) Ribbon model of RBD-C9R: The picture was generated using Pymol [20] with the coordinate of the PDB ID (6M0J) [3]. The tag region, shown in green, was generated using Modeller [21]. (**D**) SDS PAGE expression analysis: A1-soluble fraction of RBD-C9R, A2-pellet of RBD-C9R, B1-soluble fraction of RBD, B2-pellet fraction of RBD. (**E**) RP-HPLC elution profile of recombinant RBD-C9R showing the presence of a single peak indicated by an arrow. (**F**) RP-HPLC elution profile of RBD showing the presence of multiple broad peaks shown by arrows.

At all temperatures tested, the untagged RBD was expressed in the inclusion body, whereas RBD-C9R was in the soluble fraction at 16 °C (Figure 1D). The total expression level of RBD-C9R at 16 °C in LB was 6.25 µg/5 ml as estimated from the SDS page gel (Figure 1D), five times higher than for the untagged RBD expressed in the inclusion body. The ability of the C9R tag to increase protein expression is not well understood but is in line with previous observations with EFGR-ECD-III [14], TEV [16]. One-liter expression of RBD-C9R was carried out using the T7 shuffle cell line at 16 °C in LB, and RBD-C9R was purified from the soluble fraction by denaturing Ni-NTA chromatography. The His-tagged protein did not bind to the nickel resin and all the proteins appear in the flow-through fraction. One possible reason that might explain this phenomenon is the inaccessibility of the tag, which is usually the result of the tag being buried inside the protein’s three-dimensional conformation upon folding [19]. Hence, the protein was purified under denaturing conditions and further refolded upon dialysis against RO water at 4 °C for 18 h. The soluble protein was further purified using RP-HPLC. The elution profile of the RP-HPLC showed a single sharp peak (Figure 1E), suggesting the presence of a single disulfide-bonded species. In contrast, untagged RBD showed multiple broad peaks indicating a mixed population of protein with different disulfide bond pairing (Figure 1F). The final yield of RBD-C9R from the 1L culture and after RP-HPLC purification was 2 mg/L culture, whereas it was 0.3 mg/L for RBD without the C9R tag purified from the inclusion body.

### 2.3. Biophysical Characterization of RBD-C9R

**Figure 2:**
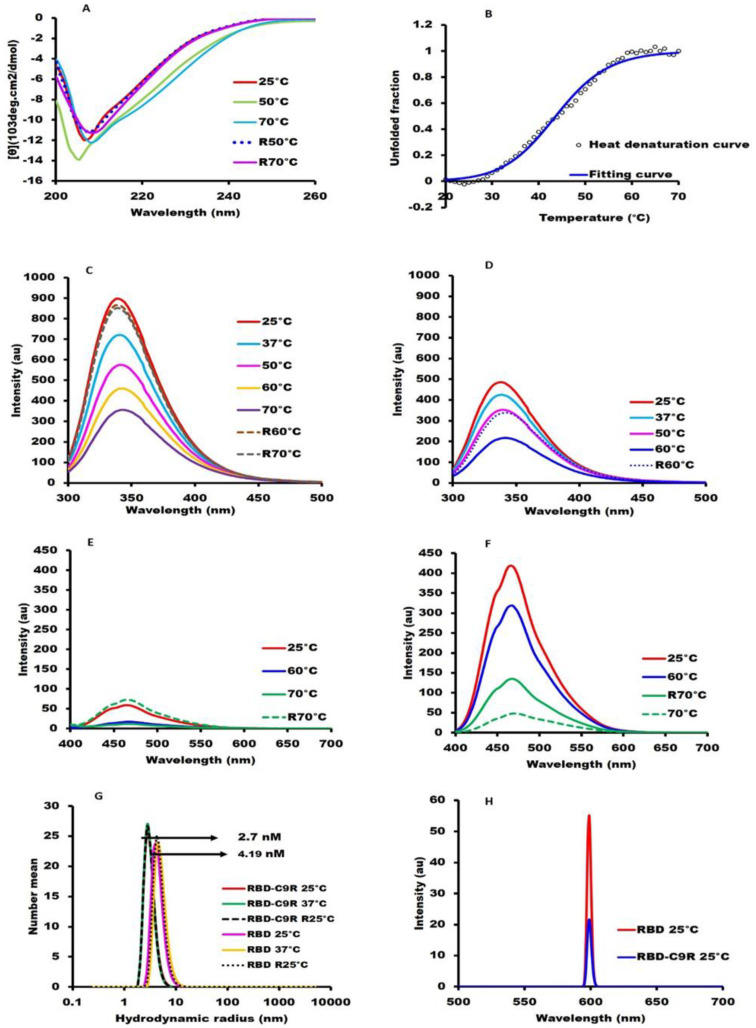
Biophysical and spectroscopic characterization: All spectroscopic measurements were performed at a protein concentration of 0.3 mg/mL in 10mM Hepes buffer, pH 8.0. (**A**) Far-UV CD spectrum of RBD-C9R (200–260 nm). (**B**) The melting temperature (*T*_m_) of RBD-C9R monitored by CD at 220 nm, the raw data are shown with open circles (ο). (**C**) Tryptophan fluorescence spectra of RBD-C9R, (**D**) Tryptophan fluorescence spectra of RBD. (**E**) ANS fluorescence spectra of RBD-C9R, (**F**) ANS fluorescence spectra of RBD, (**G**) Dynamic Light Scattering, and (**H**) Static Light Scattering. Line symbols are explained within the panels.

The far UV-CD spectrum (200–260 nm) of RBD-C9R at pH 8.0, displayed a negative peak at 208 nm, characteristic of β-sheet (Figure 2A). The secondary structure content computed using BestSel [22] was 17.4% (19.1%) α-helix and 29.2% (29.0%) β-sheet, in line with the crystal structure (PDB-ID: 6M0J data inside the parentheses) [5]. CD spectra were measured at temperatures ranging from 25 °C to 70 °C with 10 °C increments and then at 25 °C after cooling. The CD spectra indicated a reversible thermal denaturation (Figure 2A).

Cooperativity was shown by monitoring the thermal denaturation curve using CD at 220 nm (Figure 2B). However, the denaturation curve was not fully reversible, possibly because of heat-exposure-induced aggregation [23]. The CD spectrum of RBD (Figure S2) without a C9R tag was different from that of the tagged RBD with a secondary structure content of 4.6% (19.1%) α-helix and 34.1% (29.0%) β-sheet, and no thermal denaturation was observed.

The tryptophan fluorescence intensity of the C9R tagged RBD (Figure 2C) was 50% stronger with an emission maximum at 339 nm than that of the untagged RBD, indicative of the well-folded structure of RBD-C9R. Tryptophan fluorescence of the tagged variant at 70 °C indicated a 16 nm red-shift (355 nm) and a 3.6-fold decrease in fluorescence intensity (Figure 2C) typical of a change observed during protein unfolding [24,25]. ANS fluorescence, which increases upon binding to partially exposed hydrophobic pockets and is indicative of a molten-globule state, was much stronger for untagged RBD (Figure 2F) than for C9R tagged RBD (Figure 2E), suggesting that the untagged RBD might be in a loosely-packed molten-globule-like state [26], whereas RBD-C9R is well-folded.

**Figure 3:**
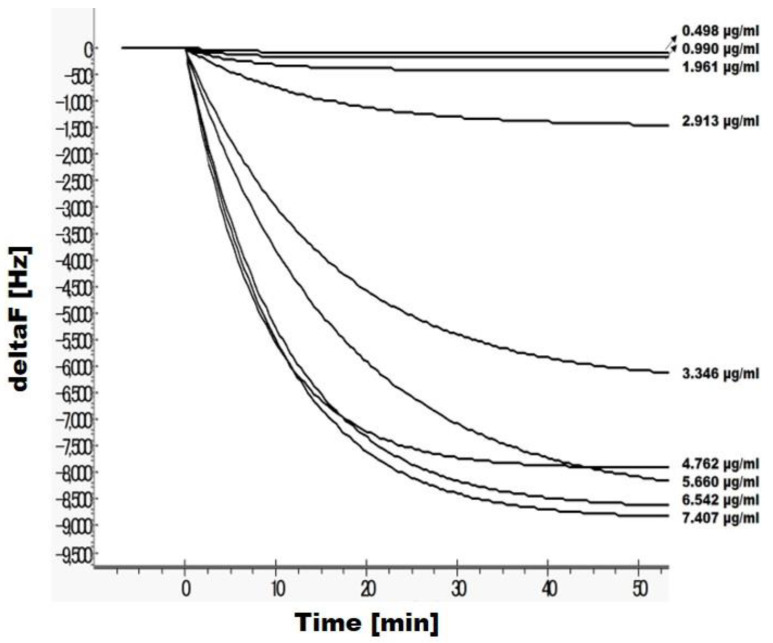
QCM analysis of the RBD-C9R binding with hACE2. Injection plot for RBD concentrations. RBD proteins concentration are indicated in panel.

The extent of solubility and the particle size of purified RBD with a C9R tag was assessed by DLS and SLS. The SLS intensity of the tagged variant was low, indicating the absence of aggregation. The SLS intensity of the untagged RBD at pH 8.0 was 8.57 times higher than the tagged variant, indicating the presence of aggregates (Figure 3G). The hydrodynamic radii [*R*_h_] measured by DLS were 2.19 nm for RBD-C9R and 4.70 nm for the untagged RBD (Figure 2H), in line with the above SLS results.

### 2.4. ACE2 Binding Activity and Immunogenicity of RBD-C9R

The binding of human ACE2, the functional receptor of RBD-C9R, was assessed by QCM analysis. ACE2 was immobilized on the QCM sensor chip and the QCM resonance signal increased in an RBD-C9R concentration-dependent manner with a dissociation constant (*K*_D_) of 1.80 × 10^−7^ M, which confirmed the proper folding of the tagged RBD-C9R (Figure 3).

A null-control experiment using BSA and BPTI yielded a negligible response and did not affect the interaction with ACE2, confirming the specificity of the binding event.

**Figure 4:**
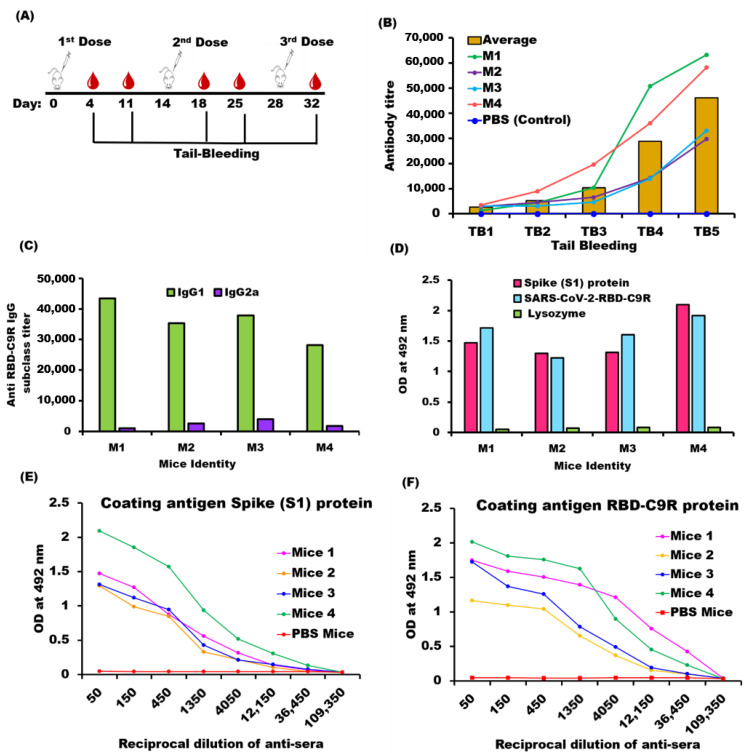
Immunogenicity of RBD-C9R. (**A**) Immunization scheme: A group of four mice was immunized with 0.3 mg/mL of RBD-C9R in 10 mM tris buffer without adjuvant. Up to three doses were injected biweekly, and tail bleed (TB) samples were collected three days after injection for monitoring the IgG titer by ELISA. M-indicates the mice’s identity, and TB indicates the tail bleeding number (once a week). (**B**) RBD-C9R IgG titer: IgG detection by ELISA was performed using the tail bleeding (TB) sera. Each circle indicates the individual mice titer, and the bars show the average titer (calculated over all four mice). (**C**) IgG sub-class (IgG1, IgG2a) Determination: Tail bleeding 5 (TB-5) serum sample was used for ELISA. (**D**) Recognition of mammalian expressed spike protein by RBD-C9R antisera: The binding of TB5 serum was measured using mammalian expressed spike (S1) protein as coating antigen and RBD-C9R as coating antigen for comparison. The plates were titrated using the mice antisera raised against RBD-C9R. The binding specificity of the antisera was determined with plates coated with lysozyme, as a control. The bar symbols are explained within the panel. (**E**) Absorbance at 492 nm vs. the reciprocal of antisera dilution against native Spike (S1) protein. (**F**) Absorbance at 492 nm vs. the reciprocal of antisera dilution against RBD-C9R (legends are given in the panel).

The RBD-C9R antisera produced by a mice immunization experiment without any adjuvants (Figure 4A) were measured every week by ELISA using RBD-C9R as the coating antigen (Figure 4B). The high IgG titers showed that the recombinant protein was immunogenic.

The IgG subclass (IgG1 and IgG2a) measurements were used to evaluate the type of immune response induced by the recipient (mouse) upon antigen immunization. A high IgG1/ IgG2a ratio indicates a Th2-type humoral immune response, whereas a low IgG1/IgG2a ratio indicates a Th1-type cellular immune response [27]. Thus, RBD-C9R induced a Th2-dependent immune response (Figure 4C). In addition, RBD-C9R antisera also interacted strongly with the mammalian cell-expressed spike (S1) protein. The binding strength, as measured by the absorbance at 492 nm, was on par with values measured by coating with the *E. coli* expressed RBD-C9R (Figure 4D, Table S1), confirming that the antisera were raised against native epitopes on the spike protein.

## 3. Discussion

The SARS-CoV-2 spike protein, RBD, is currently produced in eukaryotic cell expression systems, such as mammalian cells, yeast cells, and baculovirus–insect cells [28,29], most likely because of the presence of multi disulfide-bonds and post-translational modification. However, the production time in eukaryotic cells is long and the yield is moderate resulting in a high production cost, which does not meet the demands of therapeutic and clinical development. *E. coli* expression would be advantageous for its low production cost and scalability. Studies suggest that the RBD of the SARS-CoV S protein expressed by *E. coli* without glycosylation could provide protective immunity [30]. In the *E. coli* expression system, RBD is not expressed in a soluble form and it is difficult to refold the RBD from the insoluble fraction owing to its four disulfide bonds, which necessitates the use of expensive refolding aids [31,32]. Combining our SEP tag with low-temperature expression in the T-shuffle strain is effective for yielding soluble RBD as discussed in the next paragraphs.

The cytoplasm of wild-type *E. coli* strains is not suitable for the production of multi disulfide-bonded proteins because of its reducing cytoplasmic environment and the lack of the molecular machinery to break non-native disulfide bonds and reform native ones [33]. Despite being engineered for a more oxidative environment, the expression of multi disulfide-bonded proteins in *E. coli* strains such as Rosetta and Origami sometimes results in aberrant disulfide bondings, and the protein is expressed in the inclusion body from where it has to be refolded [34,35]. However, T7-SHuffle *E. coli* cells are engineered to contain the trxB- and gor-mutations, which alter the redox state for catalyzing the formation of disulfide bonds, and the DsbC (disulfide bond isomerase) gene, an oxidoreductase chaperone [36]. T7-SHuffle cells thus enable the oxidative folding of multi-disulfide bonds and can reshuffle the aberrant disulfide bonds of proteins expressed in its cytoplasm through DsbC isomerase [37].

Unfolded and misfolded proteins with reduced cysteines or non-native disulfide bonds have low solubility and tend to aggregate [38]. Though under an ideal infinite dilution condition, a protein tends to refold spontaneously into its native structure [39], in practice, protein folding occurs at finite concentration and thus in competition with aggregation. The SEP-tag increases the solubility of a protein in both the folded, as well as the misfolded, unfolded states through repulsive electrostatic interactions [11,14]. We thus hypothesize that the ability of the SEP-tag to maintain the unfolded and misfolded proteins in the soluble fraction provides time for the protein to refold into the native state and for the potential reshuffling of non-native disulfide bonds through cysteine isomerase or controlled redox. Further lowering the expression temperature to 16 °C slows down the protein production machinery, thereby aiding the protein folding process. Such a scenario would explain the reason for RBD-C9R expressed in the supernatant whereas RBD is in the inclusion bodies. Thus, the SEP-tag works best in the T7-shuffle cell line for expressing the multi-disulfide bonded protein in the supernatant (Figure 1D).

Analytical RP-HPLC is a relatively fast and reliable method for analyzing the formation of SS bonds [40,41], and the difference in the retention time can resolve different disulfide bonding patterns in a multi-disulfide bonded protein [16,40,41]. The RP-HPLC elution profiles of the oxidized RBD-C9R expressed in the supernatant of T7 shuffle showed a single peak, suggesting that all of the protein had folded into a single species of S–S bond pairing. Of note, the retention time of the oxidized protein differed from that of the fully reduced form, which also appears as a single peak, by approximately 2 min (Figure S1A,B).

Overall, spectroscopic and biophysical analysis of the RP-HPLC-purified RBD indicated the folded properties of RBD-C9R. Circular dichroism and fluorescence demonstrated a reversible thermal denaturation and the CD denaturation curve showed cooperativity with an apparent *T*_m_ of 43.6 °C (though aggregation made it non-reversible), which is a biophysical hallmark of a folded protein. RBD-C9R binding with ACE2 further corroborated RBD’s native-like state, as assessed by quartz crystal microbalance analysis (Figure 3). To date, the lyophilized RBD-C9R was kept at −30 °C for several weeks and was easily solubilized in MQ water, demonstrating its stability and storability.

In conclusion, the C9R-tag enabled the *E. coli* production of soluble RBD on a milligram scale. Despite owing to its limitations for the lack of glycosylation, our *E. coli*-produced RBD elicited an immune response that produced antisera recognizing the glycosylated spike (S1) protein expressed in mammalian cells, strongly suggesting that native-like epitopes are displayed on the RBD-C9R’s surface. We believe that our strategy, where we combine the capability of T7 shuffle cells for oxidizing and reshuffling disulfide bonds and the ability of the SEP-tag to maintain the solubility of unfolded proteins, is a straightforward technique for producing multi disulfide-bonded proteins in *E. coli*.

## 4. Materials and Methods

### 4.1. Plasmid Constructs

A synthetic gene encoding RBD (UniProt ID P0DTC2) was designed with codon optimization for *E. coli*. The cysteine present at the 538th amino acid position is replaced with alanine, to remove the free cysteine. A SEP-tag, C9R, consisting of three repeated blocks of three arginine residues preceded by a glycine (GR3)3 was introduced at the C-terminus of the untagged RBD using a glycine linker between RBD and the C9R tag.

### 4.2. Protein Expression and Purification

The recombinant plasmids were individually transformed into *E. coli* T7 Shuffle cells. For small-scale expression check, the cells containing recombinant plasmids were pre-cultured in 5 mL LB containing 50 μg/mL ampicillin and incubated overnight at 30 °C with shaking at 220 rpm. Then, 100 μL of cell pre-cultures was transferred into a 5 mL LB medium with antibiotics. The cell cultures were incubated with shaking at 30 °C until the cell density reached OD600 ≈ 0.6. Gene expression was induced by the addition of 0.25 mM IPTG and thereby incubating with 220 rpm shaking at 16 °C for 16–18 h and 30 °C for 6 h respectively. The protein expression was analyzed by SDS gel electrophoresis.

For large-scale expression, cells were pre-cultured overnight at 30 °C in a 35 mL LB medium with 50 μg/mL ampicillin. The pre-culture was transferred into a 1000 mL LB medium and incubated at 30 °C with 120 rpm shaking until the O.D. at 600 nm reached 0.6. After the addition of 0.25 mM IPTG, *E. coli* cells were grown further for 16–18 h at 16 °C, and cells were harvested by centrifugation at 8000 rpm (9422× *g*; Hitachi himac CF16RX centrifuge with a T9A31 rotor) at 4 °C for 20 min. The cell pellets were resuspended in lysis buffer (50 mM Tris-HCl pH 8, 150 mM NaCl), and disrupted by sonication in buffer (50 mM Tris-HCl pH 8, 1% NP-40 (*v/v*), 0.1% Deoxycholic acid (*w/v*) and 5 mM EDTA) and soluble protein extracts were obtained after centrifugation at 8000 rpm for 20 min.

6M Solid GuHCl was added to the supernatant solution. After centrifugation at 8000 rpm, 4 °C for 20 min and filtration through a 0.2 µm filter, the 6 × histidine-tagged protein was purified by using denaturing open nickel-nitrilotriacetic acid (Ni-NTA) (Wako, Japan) chromatography. The column was washed thrice with the wash buffer (6 M GuHCl, 50 mM Tris-HCl pH 6.8), and proteins were eluted with the elution buffer (6 M GuHCl and 10% Acetic acid). GuHcl was removed by 18 h dialysis against Reverse Osmosis (RO) water (four times exchange of the outer solution) at 4 °C (The molecular weight cut-off [MWCO] of the dialysis membrane was 14000). The sample was then centrifuged at 8000 rpm, 4 °C for 20 min, and the supernatant was separated from the debris. The protein was again dissolved in Milli-Q (MQ) water, lyophilized, and stored at −30 °C in a powder form until use.

### 4.3. Analytical Reverse-Phase High-Performance Liquid Chromatography (RP-HPLC)

The proteins were analyzed by reverse-phase (RP) high-performance liquid chromatography (HPLC; Shimadzu, Kyoto, Japan) using an Intrada 5WP-RP column (Imtakt, Kyoto, Japan) and absorbance at 220 nm was used to monitor the HPLC runs. Solution A (MilliQ-water + 0.1% trifluoroacetic acid (TFA)) and Solution B (Acetonitrile + 0.05% TFA) were used as a mobile phase with a flow rate of 1 mL/min and a column temperature of 30 °C. The Analytical RP-HPLC analysis was performed using a 500 µL aliquot supplemented with acetic acid at a final concentration of 10% (*v/v*), and filtered with a 0.20 µm membrane filter to remove any aggregates. The reduced form of the protein was prepared by incubating the sample at pH 8.0 with 100 mM DTT for one hour at 25 °C, and the RP-HPLC analysis was performed as mentioned above.

### 4.4. Spectroscopic Measurements

We measured the biophysical properties of the proteins in Hepes buffer, pH 8.0 at a protein concentration of 0.3 mg/mL, which is the concentration we used for the immunogenic studies. Stock solutions were prepared by dissolving the protein in MQ. The stock solutions were centrifuged at 20,000× *g* for 20 min at 4 °C and filtered with a 0.20 μm membrane filter (MDI Membrane Technology, Gurugram, India) to remove any aggregates. The final samples were prepared by diluting the stock solutions to a final protein concentration of 0.3 mg/mL in 10 mM Hepes buffer (pH 8.0). Far-UV circular dichroism (CD): CD was measured using a 2 mm path-length quartz cuvette in a continuous scanning mode. For each measurement, three scans were accumulated from 200 to 260 nm wavelength at temperatures from 25 °C to 70 °C with 10 °C increments. Reversibility was assessed by measuring the spectrum after cooling the sample to 25 °C. The secondary structure content was calculated using BeStSel [20]. Thermal stability was measured with a 1cm path-length quartz cuvette at a protein concentration of 0.15 mg/mL in 10 mM Hepes buffer (pH 8.0) at a 1 °C/min scan rate and monitored between 20 °C and 65 °C at 220 nm. The midpoint temperatures (*T*_m_) were computed by the least-square fittings of the experimental data assuming a two-state model and using Origin 6.1 J. Tryptophan and ANS fluorescence: For ANS fluorescence measurements, 8-anilino-1-naphthalene-sulfonic acid (ANS) (Wako, Japan) solution was added to the protein solution at a final concentration of 20 µM and incubated at 25 °C for 5 min in the dark before measurement [18]. Trp and ANS fluorescence spectra with λex at 295 nm and 380 nm, respectively, were measured on an FP-8500 spectrofluorometer (JASCO, Tokyo, Japan) using a quartz cuvette with a 3 mm optical path length. The sample’s temperature was increased from 25 °C to 70 °C and finally decreased back to 25 °C to assess reversibility. Light scattering: Dynamic light scattering (DLS) measurements were performed at 25 °C, 70 °C, and reverse 25 °C for pH 8.0, using a polystyrene cuvette with a Zeta-nanosizer (Malvern, UK). The hydrodynamic radius [*R*_h_] was determined from the size distribution using the Stokes–Einstein equation and averaged over three individual measurements. Static light scattering (SLS) measurements were performed under the same conditions at a wavelength of 600 nm with an FP-8500 spectrofluorometer using a quartz micro-cuvette with an optical path length of 3 mm.

### 4.5. Binding Activity by Quartz Crystal Microbalance Analysis

The interaction of RBD-C9R to human ACE2 protein was measured with a Single-Q Quartz Crystal Microbalance (AS-ONE Bioscience, Osaka, Japan) molecular interaction analyzer. According to the manufacturer’s protocol, human ACE2 protein-produced in the mammalian HEK293 cell (Bioworld technologies, Bloomington, MN, USA) was dissolved at 100 µg/mL in MQ. 10 µL were applied onto a Single-Q sensor chip on which ACE2 was immobilized by using the indirect DTDP (3,3′-Dithiodipropionic acid) method with NHS (N-Hydroxysuccinimide)/EDC (1-(3-Dimethylaminopropyl)-3ethylcarbodiimide, hydrochloride). After immobilization, the single-Q sensor chip was docked into the instrument and stabilized under a continuous flow (25 μL/min) of Hepes running buffer (10 mM, pH 7.4). After the baseline stabilized (the resonant frequency drift being less than 0.2 Hz/min), 10 μL of the analyte (10 µg/mL RBD) added to the 500 μL of 10 mM Hepes running buffer, was injected over the ACE2 immobilized sensor chip surface, allowing for interaction between the immobilized ACE2 and the RBD antigen. The frequency shift (Δf) resulting from the association on the sensor chip surface was recorded using the inbuilt-Single-Q software (AS-ONE Bioscience, Osaka, Japan). The analyte samples were injected repeatedly until there was no increase in the signal. The data were analyzed by fitting the sensorgram curves using the evaluation software provided with the single-Q QCM instrument.

### 4.6. Mice Immunization and Measurement of Serum IgG Level

Six-week-old female mice (Jcl: ICR, CLEA, Shizuoka, Japan) were used for the immunization experiments. Each of the four mice were injected subcutaneously biweekly for up to six weeks with 100 μL of 0.3 mg/mL RBD-C9R in 10 mM Tris, pH 7.4. No adjuvant was used in the experiments. A control mouse was immunized with PBS using the same protocol. For monitoring the IgG generation, tail-bleed (TB) samples were collected three days after each injection. The antisera’s IgG, IgG1, IgG2a level was assessed individually by ELISA using RBD-C9R as coating antigen. SARS-CoV-2-C9R antisera were applied to the PBS-washed wells at an initial dilution of 1:50 for tail-bleed samples. Plates were then incubated at 37 °C for 2 h. After washing the plates thrice with PBS-0.05% Tween-20, each well was filled with 100 μL of anti-mouse IgG HRP conjugate (Thermo Fisher Scientific, Waltham, MA, USA) at a 1:3000 dilution (or its subclasses-IgG1, 1:25,000 dilution /IgG2a, 1:3000 dilution) in 0.1% BSA-PBS-Tween-20 and incubated at 37 °C for 1.5 h. As a substrate, OPD (o-phenylenediamine dihydrochloride) was added. Color intensities were measured at 492 nm using a microplate reader (SH9000 Lab, Hitachi High-Tech Science Co., Tokyo, Japan) immediately after stopping the reaction with 1 N sulfuric acid (50 µL/well). Finally, titers were calculated using a power fitting model, and values were averaged over the number of mice (n).

The binding of soluble RBD antisera to full-length spike protein (S1) expressed in mammalian cells was tested by ELISA. The plate was coated separately with 100 µL of 2.5 µg/mL of the mammalian expressed full-length spike S1 protein (Invivogen, San Diego, CA, USA) and Lysozyme (Control) in 1× PBS (pH 7.4), and the following steps are mentioned above. All of the experiments were performed in compliance with TUAT’s regulations and Japanese governmental guidance on animal experimentation.

## Data Availability

The data presented in this study are available on request from the corresponding author.

## Supplementary Materials

The following supporting information can be downloaded at: https://www.mdpi.com/article/10.3390/ijms23031703/s1.
