# Supplementary material for: A Multi-Disulfide Receptor-Binding Domain (RBD) of the SARS-CoV-2 Spike Protein Expressed in E. coli Using a SEP-Tag Produces Antisera Interacting with the Mammalian Cell Expressed Spike (S1) Protein
Source: Int J Mol Sci. 2022 Feb 1;23(3):1703. doi: 10.3390/ijms23031703 (PMC8835783; doi:10.3390/ijms23031703)
Supplement: Supplementary file 1 [file ijms-23-01703-s001.zip › ijms-1484071-supplementary.pdf]

Supplementary Information

Supplementary Figures

Figure S1

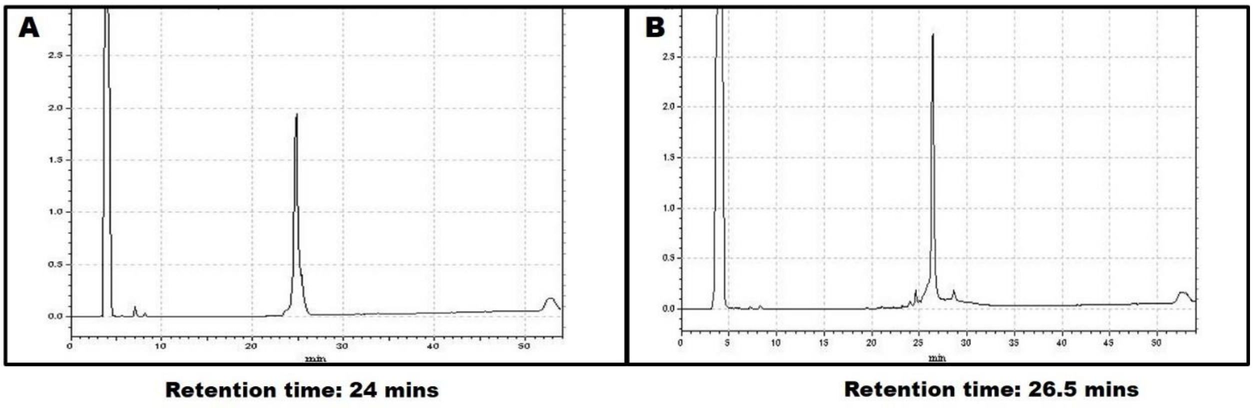

Figure S1: RP-HPLC purification: Elution profile of SARS-CoV-2-C9R (A) and reduced SARS-CoV-2-C9R [reduced with 100mM DTT].

Figure S2

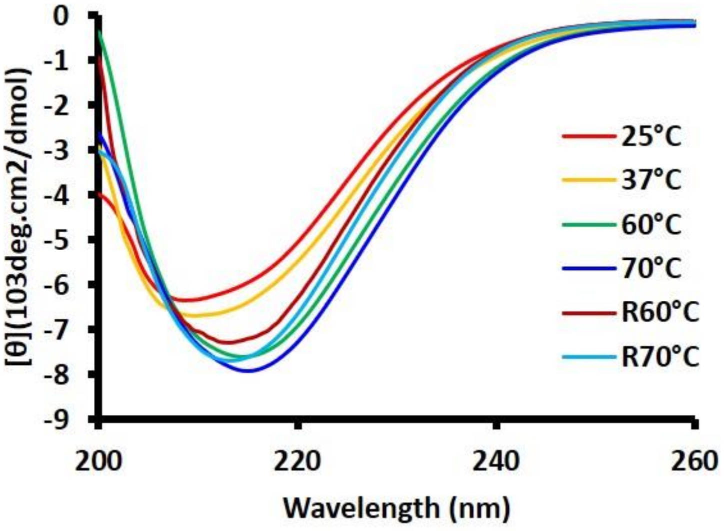

Figure S2: The secondary structures of SARS-CoV-2 were analyzed using far-UV CD region (200–260 nm) at a protein concentration of 0.3 mg/mL in 10mM Hepes buffer, pH 8.0.

Table S1: Estimation of EC50 values based on the reciprocal dilutions of the 5th tail bleeding RBD-C9R serum and immobilized antigens (Native spike (S1) protein and RBD-C9R protein).

	EC 50 Value			
	Mice 1	Mice 2	Mice 3	Mice 4
Log 10 Reciprocal serum dilution (Spike S1 protein as coating antigen)	2.68	2.70	2.87	3.00
Log 10 Reciprocal serum dilution (RBD-C9R protein as coating antigen)	3.98	3.24	2.98	3.59
